# INVOLUNTARY JOB LOSS AND GENDERED HEALTH OUTCOMES IN MIDDLE AGE AND LATER LIFE: A LIFE COURSE ANALYSIS

**DOI:** 10.1093/geroni/igac059.3007

**Published:** 2022-12-20

**Authors:** Qian Song, Caitlin Connelly

**Affiliations:** Universiy of Massachusetts Boston, Boston, Massachusetts, United States; UMASS Boston, Boston, Massachusetts, United States

## Abstract

Job loss is associated with a range of negative health outcomes. However, the causality of job loss and poor health is unclear, due to issues of endogeneity and reverse causality (i.e., adverse health causing job loss). We also need a better understanding of the long-term effects of job loss, and how the life stage in which job loss occurred yields different health outcomes for women and men in later life. To address these gaps, we take advantage of a quasi-experimental setting — the policy-driven layoffs of the State-Owned-Enterprises (SOEs) during the 1990s – mid-2000s in urban China. Using the life history survey of the China Health and Retirement Longitudinal Study 2014 and the 2015 wave, we examine the long-term effects of job loss on self-rated health (scored 1 – 5) of individuals in middle age and later life (aged 45+). After controlling for individual childhood health, life-time work history, as well as demographics and socioeconomic status, results from linear regressions show that overall, job loss from SOEs reduces self-rated health by 0.12 (p < .05) for both women and men. However, job loss hurts females’ self-rated health the most when job loss occurred in early-life, a life stage in which childbearing and child caregiving were most intensive (aged 35 or earlier; β = -0.24, p < .05), whereas males’ self-rated health is hardest hit if job loss occurred in mid-life (aged 36 – 45; β = -0.35, p < .01). The results suggest that involuntary job loss produce gendered health outcomes due to gendered life courses.

